# Pseudopemphigoid: A systematic review

**DOI:** 10.1016/j.jdin.2024.07.019

**Published:** 2024-08-23

**Authors:** Brandon Levian, Syedah S. Hussaini, David T. Woodley, Michael Kasperkiewicz

**Affiliations:** a Department of Dermatology, Keck School of Medicine, University of Southern California, Los Angeles, California; b Scientific Group of Dermatology, Jagiellonian University Medical College, School of Medicine in English, Cracow, Poland

**Keywords:** mucous membrane pemphigoid, ocular pemphigoid, pseudopemphigoid

*To the Editor:* Pseudopemphigoid, first described by Patten et al in 1976,^1^ is a rare disorder that clinically mimics the immunobullous disease ocular mucous membrane pemphigoid (MMP), but typically lacks its immunological characteristics such as subepithelial linear deposition of autoantibodies/complement.^2^^,^^3^ The existing literature on pseudopemphigoid has not yet been fully evaluated by a systematic review.

This systematic review was conducted following preferred reporting items for systematic reviews and meta-analyses (PRISMA) guidelines. Literature from the inception of the database until 8 May 2024 was explored using PubMed and the keyword “pseudopemphigoid.” Inclusion criteria were peer-reviewed, English language articles about pseudopemphigoid cases. Pure reviews, guidelines, basic research studies, and articles not meeting the inclusion criteria were excluded.

We included 18 suitable papers published between 1976 and 2022 (Fig  1). These comprised 9 case series, 5 case reports, 3 interventional studies, and 1 retrospective cohort study (*n* = 202 individuals with pseudopemphigoid; 121 [59.9%] female and 81 [40.1%] male, age range 7-92 years). The most common clinical manifestations included scarring (*n* = 121, 59.9%), inflammation/conjunctivitis (*n* = 39, 19.3%), and dry eyes (*n* = 37, 18.3%). Unilaterality and bilaterality of ocular symptoms were observed in 22 (10.9%) and 30 (14.9%) individuals, respectively, whereas information about the remaining 150 (74.3%) cases was lacking. Testing for tissue-bound immunoreactants by direct immunofluorescence of the conjunctiva was either negative (*n* = 26, 12.9%) or not reported (*n* = 171, 84.7%), whereas a positive linear basement membrane zone or intercellular immunoglobulin/complement staining was found in 2 (1.0%) and 3 (1.5%) cases, respectively.^1^^,^^4^^,^^5^ While testing for circulating pemphigoid/pemphigus autoantibodies was also not reported in the majority of cases (*n* = 195, 96.5%) and 4 patients (2.0%) were negative, 3 (1.5%) patients had positive IgG/IgA antibasement membrane zone antibodies detected by indirect immunofluorescence microscopy using normal human conjunctiva as substrate.^4^ Presumed causes of pseudopemphigoid included medications (*n* = 96, 47.5%), rosacea (*n* = 30, 14.9%), atopic keratoconjunctivitis (*n* = 12, 5.9%), and conjunctival lichen planus (*n* = 12, 5.9%). The most commonly used inciting medications were topical anti-glaucoma agents such as timolol (*n* = 60, 29.7%), pilocarpine (*n* = 28, 13.9%), and brimonidine (*n* = 21, 10.4%). Management of pseudopemphigoid mostly involved withdrawing the causative medication (*n* = 22, 10.9%), topical corticosteroids (*n* = 10, 5.0%), and nonspecified immunosuppressive therapy (*n* = 10, 5.0%). Of 48 patients with reported treatment outcomes, 46 patients (95.8%) were noted to have improvement of their symptoms, while progressive disease was observed in 2 (4.2%) cases. Treatment outcomes were not described in the remaining 154 cases.

**Fig 1:**
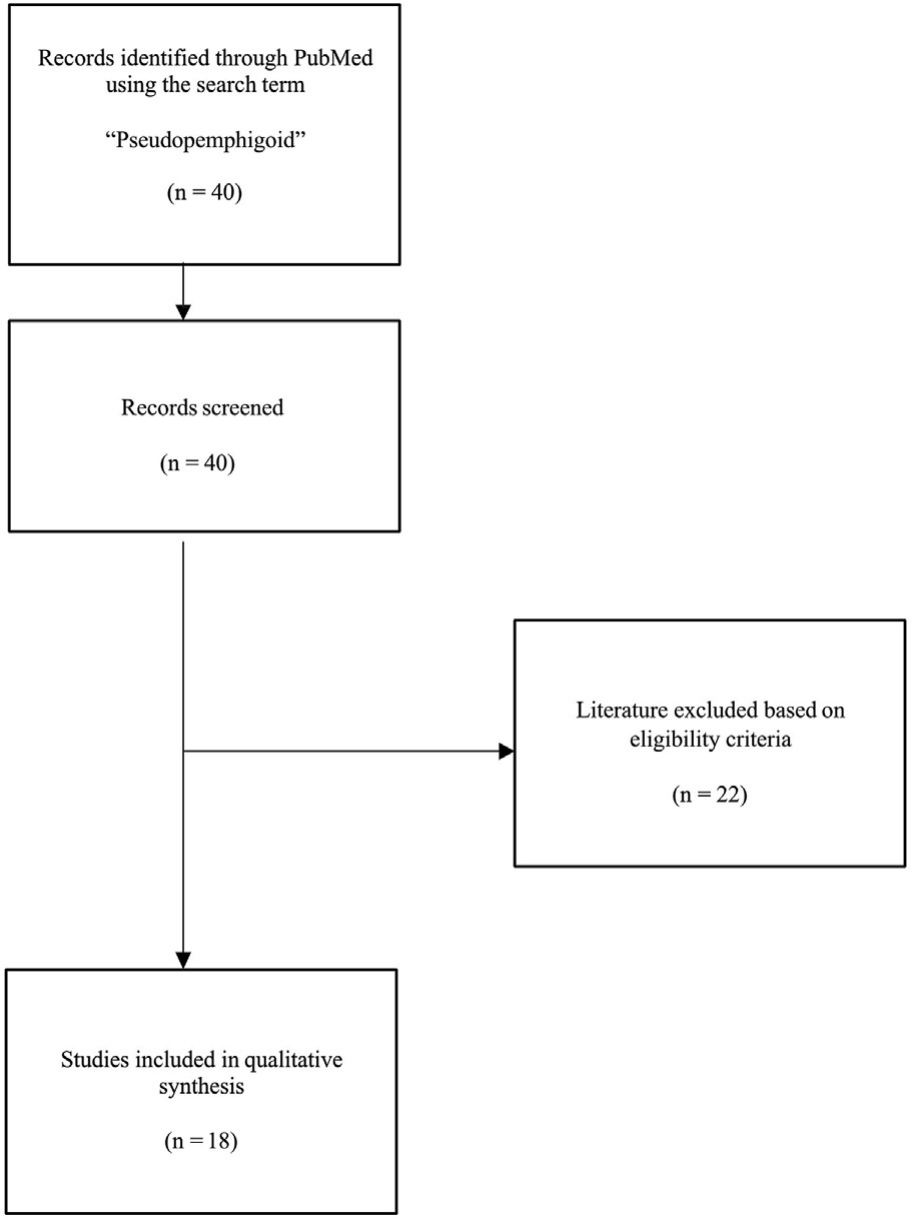
Flowchart of the article selection process.

This systematic review, involving over 200 patients, revealed several common findings associated with pseudopemphigoid: a slight female predominance, ocular scarring, disease induction by topical anti-glaucoma medications, management by withdrawing the offending drug, and a favorable disease course. Of note, a few patients had a positive pemphigoid immunopathology,^1^^,^^4^ and in the majority of cases, information about autoantibody/complement testing to rule out MMP was not available, suggesting potential misdiagnosis. Our investigation highlights the need for a thorough immunologic workup (ie, direct/indirect immunofluorescence, enzyme-linked immunosorbent assay, and/or immunoblot) to better differentiate pseudopemphigoid from ocular MMP through a close collaboration of ophthalmologists with dermatologists, considering that the latter disease is generally associated with a poorer prognosis and the requirement for systemic immunosuppression.^3^

## Conflicts of interest

None disclosed.
